# Measurement of Circulating Filarial Antigen Levels in Human Blood with a Point-of-Care Test Strip and a Portable Spectrodensitometer

**DOI:** 10.4269/ajtmh.15-0916

**Published:** 2016-06-01

**Authors:** Cédric B. Chesnais, Johnny Vlaminck, Billy Kunyu-Shako, Sébastien D. Pion, Naomi-Pitchouna Awaca-Uvon, Gary J. Weil, Dieudonné Mumba, Michel Boussinesq

**Affiliations:** Unité Mixte Internationale (UMI) 233, Institut de Recherche pour le Développement (IRD), Montpellier, France; Université Montpellier, Montpellier, France; Institut National de la Santé et de la Recherche Médicale (INSERM) Unité 1175, Montpellier, France; Infectious Diseases Division, Department of Medicine, Washington University School of Medicine, St. Louis, Missouri; Department of Parasitology, National Institute of Biomedical Research (INRB), Kinshasa, Democratic Republic of the Congo; National Onchocerciasis Control Programme, Ministry of Public Health, Kinshasa, Democratic Republic of the Congo

## Abstract

The Alere Filariasis Test Strip (FTS) is a qualitative, point-of-care diagnostic tool that detects *Wuchereria bancrofti* circulating filarial antigen (CFA) in human blood, serum, or plasma. The Global Program to Eliminate Lymphatic Filariasis employs the FTS for mapping filariasis-endemic areas and assessing the success of elimination efforts. The objective of this study was to explore the relationship between the intensity of positive test lines obtained by FTS with CFA levels as determined by enzyme-linked immunosorbent assay (ELISA) with blood and plasma samples from 188 individuals who live in a filariasis-endemic area. The intensity of the FTS test line was assessed visually to provide a semiquantitative score (visual Filariasis Test Strip [vFTS]), and line intensity was measured with a portable spectrodensitometer (quantitative Filariasis Test Strip [qFTS]). These results were compared with antigen levels measured by ELISA in plasma from the same subjects. qFTS measurements were highly correlated with vFTS scores (ρ = 0.94; *P* < 0.001) and with plasma CFA levels (ρ = 0.91; *P* < 0.001). Thus, qFTS assessment is a convenient method for quantifying *W. bancrofti* CFA levels in human blood, which are correlated with adult worm burdens. This tool may be useful for assessing the impact of treatment on adult filarial worms in individuals and communities.

## Introduction

Lymphatic filariasis (LF) is a parasitic disease that is a significant cause of disability in the developing world. About 90% of the estimated 120 million cases of LF in the world are caused by the filarial parasite *Wuchereria bancrofti*.^1^ The Global Program to Eliminate Lymphatic Filariasis (GPELF; coordinated by the World Health Organization [WHO]) aims to eliminate LF by 2020 using repeated rounds of mass drug administration (MDA). Diagnostic testing is important for each phase of this program including mapping, assessing the impact of MDA, transmission assessment surveys, and post-MDA surveillance.^2^–^4^

At present, three laboratory methods are used for diagnosing active infections with *W. bancrofti*, namely demonstration of microfilariae (mf) in night blood specimens, detection of circulating filarial antigens (CFA) released in the blood by adult worms, and detection of filarial DNA in human blood by polymerase chain reaction (PCR). Antigen testing is most widely used at this time, because it is more sensitive and convenient for detecting infection than mf testing or PCR.^2^,^5^ Two types of antigen tests are currently available: enzyme-linked immunosorbent assay (ELISA) tests performed in the laboratory, and point-of-care (POC) rapid tests. ELISA tests are based on monoclonal antibodies AD12.1 or Og4C3. They provide quantitative results^6^,^7^ but are not practical for routine use in LF elimination programs. The rapid tests include the BinaxNOW^®^ Filariasis card test (Alere, Scarborough, ME)(immunochromatography card test [ICT]), which has been used in GPELF since 2000, and the Filariasis Test Strip (FTS), introduced in 2013 by Alere, the successor company to Binax.^8^

The WHO and the manufacturer recommend qualitative reading of ICTs and FTS as either positive or negative so that the result of surveys is generally expressed as prevalence of antigenemia. In practice, the test line shows a wide range of intensity, from clear to dense.

On the basis of the relative intensities of the test (T) and control (C) lines, ICT scores have been informally used for many years, assuming a relationship between this score and the level of CFA and, possibly, with the burden of adult worms.^8^,^9^

Although visual scoring of rapid format filarial antigen tests is useful, more accurate quantitation would represent a step forward. Rapid quantitation of antigen levels could be used to assess the effect of treatment on adult worms in individuals and the impact of MDA on adult worm loads in communities without the need for ELISA. Therefore, the purpose of this study was to explore the use of a portable spectrodensitometer together with the FTS for measuring CFA levels in human blood to test whether the intensity of T-lines in positive FTS is correlated with the level of CFA.

## Materials and Methods

### Study area and subjects.

This research was performed as part of a community study in the Democratic Republic of the Congo (DRC) of the impact of semiannual mass treatment with albendazole on bancroftian filariasis. The trial was conducted in two villages (Mbumkimi and Misay) that are situated on the right bank of the Kwilu River in the Bagata Health Zone (Bandundu Province). Filarial antigen rates assessed by FTS in June 2014, before any treatment, were 32.5% in Mbumkimi and 31.6% in Misay.

We tested 188 blood samples in this study. These included 110 samples (95 with positive FTS results and 15 negative samples) collected in July 2014 before any treatment in the study communities and 78 samples (69 positives and nine negatives) during a follow-up survey in July 2015 after two rounds of MDA with albendazole, which has partial macrofilaricidal activity against *W. bancrofti* adult worms.^10^,^11^ Because the focus of the study was on quantitation of positive tests, we oversampled positive tests and selected samples with range of positivity.

The Ethical Committee for Research in the DRC (Ministry of Public Health) approved this study. Written informed consent was obtained from all adults participating in the study and from parents or legal guardians of participants younger than 18 years of age.

### The FTS and test performance.

Antigen testing was performed with Alere Filariasis Test Strips (Alere, Scarborough, ME) according to the manufacturer's instructions. This test detects a circulating filarial antigen essentially as previously described for the ICT but with a different platform.^2^,^12^ In brief, 75 μL of finger prick blood is added to a sample application pad that contains dried polyclonal antibody to CFA that has been labeled with colloidal gold. The labeled antibody binds to CFA (if it is present), blood cells are retained in the sample pad, and the labeled antigen–antibody complexes flow down a nitrocellulose strip with the plasma. The immune complexes are immobilized when they bind to a monoclonal antibody to CFA that has been striped across the nitrocellulose membrane, and this results in a positive test with a visible “T-line.” The procedural control “C-line” develops when excess labeled polyclonal antibody crosses a line that contains a secondary antibody to the immunoglobulin that was in the sample pad. Thus, samples that contain CFA produce visible T- and C-lines in the FTS, while negative samples only produce the C-line.

Antigen testing was performed during the day in study villages. Fingers were cleaned with an alcohol wipe and pricked with a microtainer contact-activated lancet (Becton Dickenson, Franklin Lakes, NJ), and 75 μL of blood was collected from the finger with a plastic micropipette included in the kit and directly placed onto the FTS sample application pad. A single trained operator read the FTS at 10 minutes. Visual Filariasis Test Strip (vFTS) results were scored semiquantitatively as previously described: negative tests with no visible test (T-) line were assigned a score of 0, tests with visible T-line weaker than the control (C-) line were assigned a score of 1, tests with a T-line that was approximately equal in density to the C-line were scored 2, and tests with a T-line darker than the C-line were scored 3.^9^

### Quantitative assessment of filarial antigenemia.

#### Spectrodensitometric measurement of FTS results (quantitative Filariasis Test Strip).

A compact portable spectrodensitometer (FD-5; Konica-Minolta Inc., Tokyo, Japan) was used to measure the intensities of the FTS C- and T-lines. The measurements were performed immediately after the visual reading, that is, 10 minutes after placement of the blood on the sample application pad. For reading, completed test strips were placed in a template produced with unused FTS that were glued onto white cardboard. The unused strips created a slot that held the used FTS in place and allowed for consistent spectrodensitometric reading of the FTS T- and C-lines. Before each measurement, readings were also made using an unused FTS (the white “target”) for calibration. The target window of the spectrodensitometer was placed successively on the C- and T-lines of the used FTS strip. This circular window (diameter: 3.5 mm) was not adapted to the size of the reaction lines. Hence, the spectrodensitometric readings reflect the entire field of view of the target window and not just the C- or T-lines. Background values from the white target were subtracted from these values to obtain ΔRed_C-line_ and ΔRed_T-line_. The quantitative Filariasis Test Strip (qFTS) ratio was defined as ΔRed_T-line_:ΔRed_C-line._ Thus, a qFTS ratio close to 0 should be theoretically analogous to a vFTS score of 0, a qFTS ratio between 0 and 1 to a vFTS score of 1, a qFTS ratio of approximately 1 to a vFTS score of 2, and a qFTS ratio > 1 to a vFTS score of 3.

#### Measurement of circulating filarial antigens by ELISA.

Finger prick blood was collected into heparinized capillary tubes and stored in a cooler. Tubes were centrifuged, and plasma was separated and stored at −20°C. The Tropbio Og4C3 ELISA kit (Cellabs, New South Wales, Australia) was used to measure CFA levels according to the manufacturer's instructions. Samples were tested in duplicate. Kit control standards (S1–S7) and a conjugate control were also tested in duplicate on each plate. According to the manufacturer's instructions, the standard control sample 1 (S1) should contain less than 10 antigen units (AU); samples with optical density (OD) values lower than the OD produced by S2 (with 32 AU) were considered to be negative. Samples with OD values between those obtained by S2 and S3 (with 128 AU) were considered to be equivocal. Samples with OD values greater or equal to the OD obtained with S3 were considered to be positive for CFA. ELISA assays were performed blindly without reference to the vFTS or qFTS results. The laboratory testing was performed in the Department of Parasitology at the National Institute of Biomedical Research in Kinshasa.

### Statistical analysis.

All reported means are arithmetic means unless otherwise specified. Differences in mean qFTS ratios and mean ELISA OD values in the different vFTS score groups were analyzed using Cuzick's trend test. We used Spearman's rank correlation coefficient to assess the associations between qFTS, vFTS, and ELISA OD values. Receiver operating characteristic (ROC) analysis was used to select cutoff values for ELISA and qFTS data. We first determined the optimal cutoff for the qFTS ratio using vFTS positivity as the reference standard. Subsequently, we did the same using Og4C3 ELISA results as the reference standard. Finally, kappa scores were calculated to indicate the degree of agreement between different antigen test methods. All statistical analyses were performed using STATA 14.0 (StataCorp, College Station, TX), and GraphPad Prism version 6.0e (GraphPad Software, Inc., La Jolla, CA).

## Results

### Description of vFTS results.

The study was performed with blood samples from 188 subjects that included 164 (87.2%) with positive vFTS results. Of study participants, 24 had a vFTS score of 0 (12.8%), 52 had a score of 1 (27.7%), 38 had a score of 2 (20.2%), and 74 had a score of 3 (39.4%).

### Description of qFTS results.

**Figure 1.**
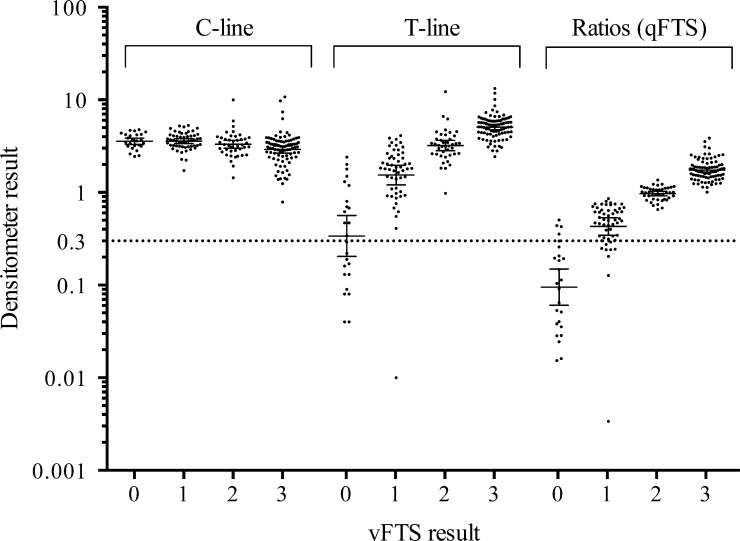
Distribution of the densitometer results for the control line (C-line), the test line (T-line), and the T/C ratios (quantitative Filariasis Test Strip [qFTS]) according to visual Filariasis Test Strip (vFTS) results. The optimal cutoff for positivity of qFTS was determined to be 0.30 (dotted line) using receiver operating characteristic analysis and vFTS results as reference standard. Solid horizontal lines represent geometric means and 95% confidence intervals.

Spectrodensitometry data for C- and T-lines and T:C ratios are shown in Figure 1

. A slight but significant decrease in C-line intensity was observed with increasing vFTS scores (11.6% decrease in mean C-line intensity for tests with vFTS scores of 3 relatively to the mean for tests with vFTS scores of 0, *P* < 0.001 by Cuzick's trend test). Mean T-line intensities increased from 0.64 (±0.14) for vFTS negative individuals to 1.89 (±0.13), 3.51 (±0.28), and 5.34 (±0.19) for test strips with vFTS scores of 1, 2, and 3, respectively (*P* < 0.001 by Cuzick's trend test). The mean qFTS ratio was 0.16 (±0.03) for samples that were negative by vFTS; qFTS ratios were 0.50 (±0.03), 0.98 (±0.03), and 1.77 (±0.05) for samples with vFTS scores of 1, 2, or 3, respectively (*P* < 0.001 by Cuzick's trend test). Although C-line intensities were slightly decreased in samples with vFTS scores of 3, we decided that qFTS ratios were better than T-line densities for presenting qFTS results, since the ratios tend to correct for variable volumes of plasma due to variable hematocrits between people present in whole blood samples added to sample application pads. There were highly significant correlations between qFTS ratios and vFTS scores when all samples were considered (Spearman's rank correlation coefficient 0.94; *P* < 0.001) and when samples collected before and after MDA were considered separately (ρ = 0.93 and 0.94, respectively; both *P* < 0.001) (Table 1).

### Filarial antigenemia ELISA results.

**Figure 2.**
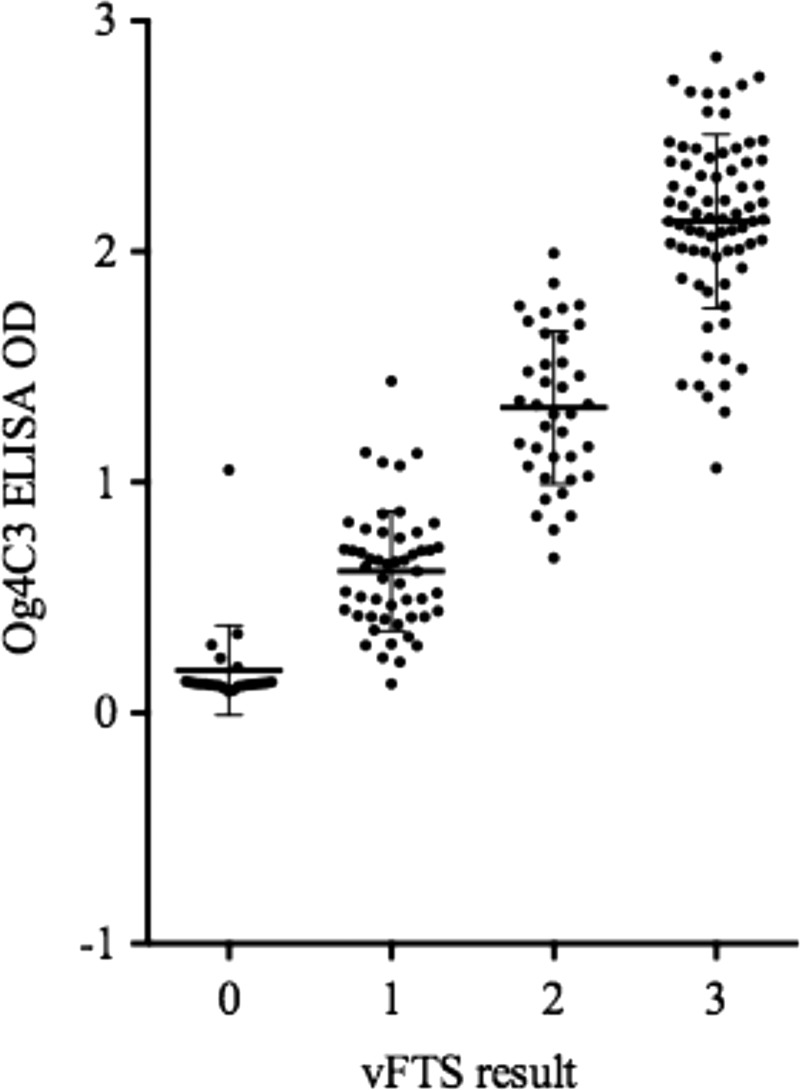
Relationship between the optical density (OD) values obtained by Og4C3 ELISA and the visual Filariasis Test Strip (vFTS) scores. Solid horizontal lines represent geometric means and 95% confidence intervals. ELISA = enzyme-linked immunosorbent assay.

Kit standards produced valid results on all five plates tested with OD values < 0.20 for the negative control standard S1 and OD values > 2.0 for the strong positive control standard S7. Plasma samples from individuals with vFTS scores of 0, 1, 2, or 3 had mean OD (± standard deviation) values 0.18 (±0.04), 0.61 (±0.04), 1.32 (±0.05), and 2.12 (±0.05), respectively (*P* < 0.001 by Cuzick's trend test) (Figure 2

).

### Agreement between results of vFTS, qFTS, and ELISA.

**Figure 3.**
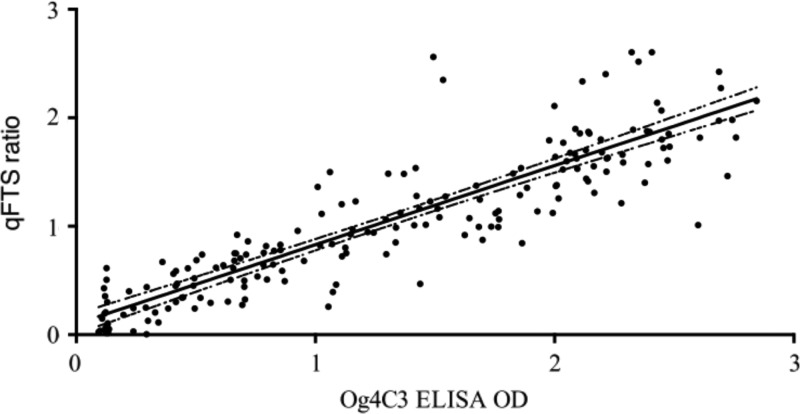
Relationship between qFTS ratios and Og4C3 ELISA OD values for all samples tested by both methods (Spearman's rank correlation coefficient ρ = 0.91, *P* < 0.001) with a fitted regression line and 95% confidence intervals. ELISA = enzyme-linked immunosorbent assay; OD = optical density; qFTS = quantitative Filariasis Test Strip.

Spearman's rank correlation coefficients between Og4C3 ELISA OD values and vFTS scores were 0.91, 0.92, and 0.90 (all *P* < 0.001) in the whole, pre-, and post-MDA populations, respectively (Table 1). Spearman's rank correlation coefficient for relationship between ELISA OD and qFTS scores was strong for the whole sample (ρ = 0.91; *P* < 0.001) (Figure 3

), and in pre- and posttreatment samples (ρ = 0.90 [*P* < 0.001] and 0.90 [*P* < 0.001] respectively) (Table 1). This suggests that qFTS is as useful as ELISA for estimating CFA levels and that albendazole treatment does not influence the correlation.

### Sensitivity analysis.

#### Selection of positive cutoff values for qFTS using vFTS results as a reference standard.

An ROC analysis compared qFTS ratios to qualitative vFTS results (positive [vFTS ≥ 1] or negative [vFTS = 0]) to determine a cutoff value for positivity using the qFTS ratio (Table 2). When the qFTS ratio cutoff was 0.30, the sensitivity for qFTS versus vFTS was 94.9% (95% confidence interval [CI]: 90.6–97.7), specificity was 84% (95% CI: 63.9–95.5), and 93.6% of the individuals were correctly classified. Using this cutoff, four individuals had false-positive results (qFTS+/vFTS−) and nine had false-negative results (qFTS−/vFTS+). All nine samples of the individuals with qFTS−/vFTS+ had vFTS scores of 1. Figure 1 illustrates that the placement of the qFTS ratio cutoff at 0.30 easily discriminated the negative from the positive vFTS results. The kappa score of 0.73 (*P* < 0.001) indicates a high level of agreement between vFTS and qFTS results.^13^

#### Selection of a positive cutoff value for qFTS using Og4C3 ELISA results as reference standard.

**Figure 4.**
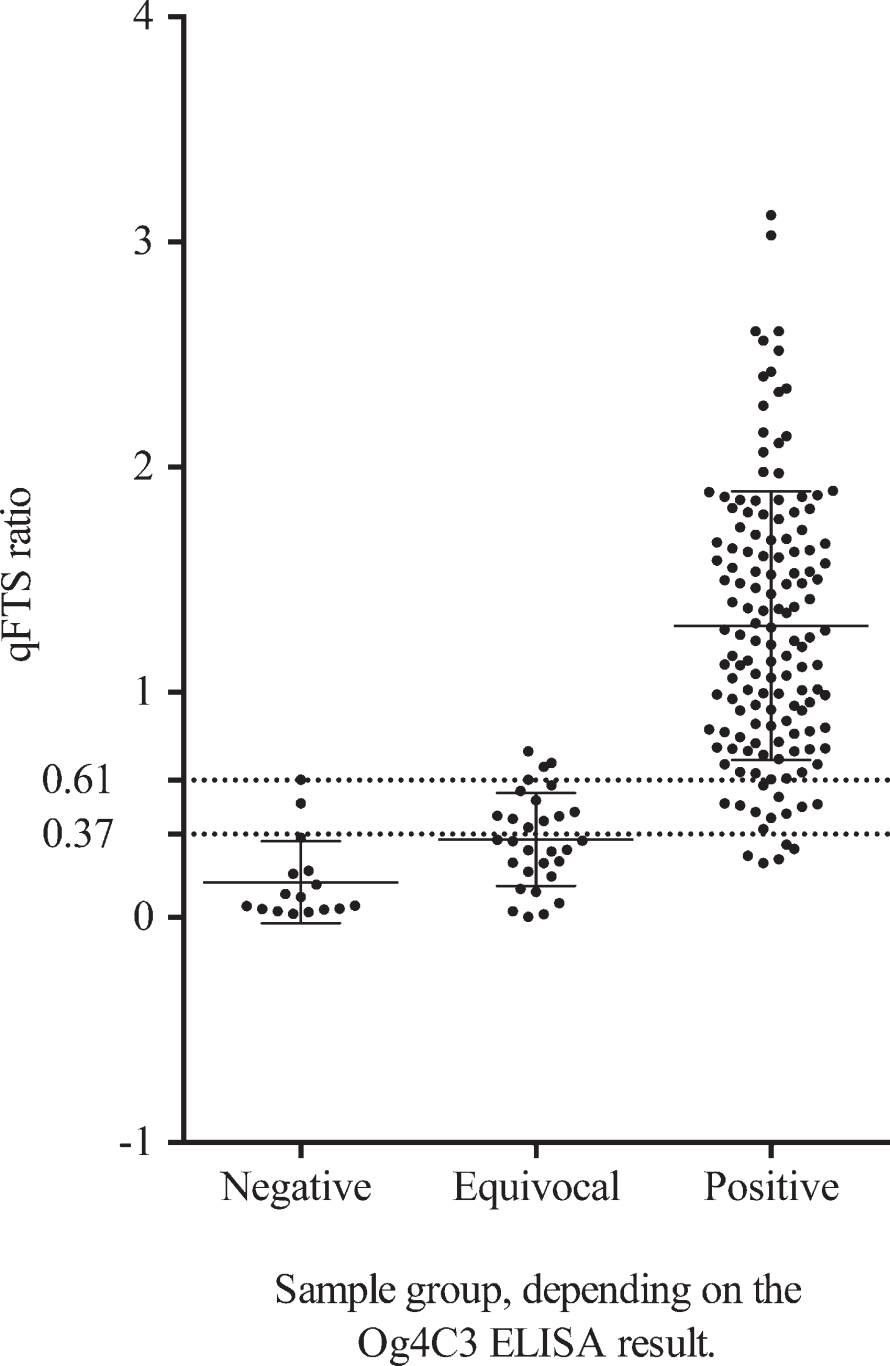
qFTS ratios of individuals that tested negative (OD < S2), equivocal (S2 < OD < S3), or positive (OD > S3) for circulating filarial antigen (CFA) by Og4C3 ELISA. The dotted lines represent the optimal cutoff values determined by receiver operating characteristic analysis when only the negative group was considered negative (cutoff = 0.37) or when both the negative and equivocal groups were considered to be negative for CFA (cutoff = 0.61). Solid horizontal lines represent geometric means and 95% confidence intervals. ELISA = enzyme-linked immunosorbent assay; OD = optical density; qFTS = quantitative Filariasis Test Strip.

Samples were sorted into different groups according to their respective OD values. The negative group contained individuals with an Og4C3 ELISA OD below the OD of the kit standard 2 (32 AU). A second group contained individuals with Og4C3 results between OD values obtained with the kit standards 2 and 3 (128 AU) that are equivocal according to the kit guidelines. A third, positive group contained individuals with ELISA ODs higher than that obtained with the kit standard 3 (positive according to the kit guidelines). Subsequently, qFTS ratios were plotted for all individuals in the different groups, and qFTS sensitivity and specificity was determined relative to the ELISA result. This yielded sensitivity and specificity values for qFTS of 87.2% (95% IC: 81.3–91.8) and 87.5% (95% CI: 61.6–98.5), respectively, when only the negative group was considered negative, and 93.5% (95% CI: 82.1–98.6) and 89.4% (95% CI: 83.2–94.0), respectively, when samples from the negative and equivocal group were considered negative. The corresponding optimal qFTS ratio cutoff values for positivity were determined by ROC analysis and were placed at an OD of 0.37 and 0.61, respectively (Figure 4

). These qFTS ratio cutoff values were higher than those obtained when vFTS results were used as the reference standard.

#### Comparison of vFTS results with Og4C3 ELISA results.

Table 3 compares vFTS results with Og4C3 ELISA results using the OD obtained by either kit standard S2 or S3 as threshold. When kit standard S2 was used as cutoff for positivity, only 29 of 52 (56%) subjects with vFTS scores of 1 had positive ELISA results. When kit standard S3 was used as cutoff for positivity (as recommended by the package insert) the sensitivity of ELISA for detecting antigen in samples with vFTS scores of 1 was even lower (10 of 52 or 19%). The S1 negative control in the Og4C3 kit was negative by vFTS, whereas the S2 in the kit was clearly positive by vFTS. These results show that vFTS (and indirectly qFTS) is more sensitive than the Og4C3 ELISA for detecting CFA.

## Discussion

WHO guidelines for the GPELF recommend use of filarial antigen tests for mapping endemic areas and for surveys to verify that countries have reached elimination targets following MDA,^14^ and the WHO has recently approved the Alere FTS for this purpose.^15^ These guidelines assume qualitative antigen testing (positive or negative), and they do not mention use of these tests for quantifying CFA. We have recently demonstrated the value of semiquantitative reading of ICTs,^9^ and this study goes one step further by using spectrodensitometry to measure the relative concentration of CFA in blood samples. This method allows the user to quantify antigen levels in the field, and it avoids the cost and laboratory infrastructure required for antigen testing by ELISA. It is very interesting that qFTS ratios were strongly correlated with vFTS scores and with OD values obtained with the Og4C3 ELISA. Since CFA levels are believed to be correlated with adult *W. bancrofti* worm numbers,^16^–^18^ qFTS ratios should be very useful for assessing the impact of treatment on adult filarial worms in individuals. Because of its low cost and simplicity, qFTS holds great promise as a practical, field-friendly tool for GPELF to measure adult worm burdens at the community level before and after MDA, much as community microfilarial load is sometimes used to monitor the impact of MDA on the community parasite reservoir in LF and onchocerciasis.^19^,^20^ Although qFTS provides a more accurate and objective reading than vFTS, qFTS was slightly less sensitive than vFTS for reading weak positive antigen tests.

A comparison of qFTS and vFTS results with Og4C3 ELISA results suggests that both the qFTS and vFTS are more sensitive than the ELISA when the S3 antigen standard is used as the cutoff for definite positivity as recommended by the manufacturer. Our results suggest that a significant proportion of samples that produce OD values in the Og4C3 ELISA between those produced by the S2 and S3 kit standards are true positives. Low ELISA OD values for some samples that were positive by vFTS also indicate a lower sensitivity of the ELISA. Hence, filarial infection rates based on CFA in the blood measured by Og4C3 ELISA will tend to be underestimated.

Some inconsistencies were detected between results obtained by qFTS and vFTS; specifically, there was some overlap between qFTS ratios of samples with vFTS scores of 0 and 1. Although this could indicate that some vFTS readings were falsely positive, we think it is more likely that this overlap is due to imperfect or inconsistent performance of the spectrodensitometer when T-lines are very light. With the methods used in this study, vFTS seems better than qFTS for classifying samples as positive or negative when antigen levels are very low. It should be noted that the spectrodensitometer used in this study was designed for use in the printing industry and not optimized for reading FTS. Our homemade template for positioning FTS strips may have introduced minor reading errors. However, our study has provided an interesting proof of principle for quantitative reading of filariasis POC tests. A purpose-built instrument might produce better results. Such instruments already exist but they are very expensive. However, a recent review describes cellphone applications that have been used to read POC tests for several analytes and markers of infectious diseases such as malaria and human immunodeficiency virus,^21^ and we look forward to further development of this approach.

We noticed a slight decrease in C-line density (by about 12%) for samples with vFTS scores of 3. This is a minor limitation, because it will tend to increase qFTS ratios for strongly positive samples. The sample application pad in the FTS contains an excess amount of labeled antibody to CFA, and the intensity of C-lines was the same for samples with vFTS scores of 0, 1, or 2. However, samples with high levels of CFA resulted in strong T-lines (vFTS score of 3) with less labeled antibody available to produce the C-line. This did not significantly affect the main findings of our study. To check on this, we have recalculated the correlation between qFTS ratios and Og4C3 levels after increasing C-line intensities by 12% for samples with FTS scores of 3. The correlation was barely affected by this adjustment (ρ = 0.90; *P* < 0.001). To our knowledge, reduced intensity of C-lines with strongly positive samples has not previously been described for the FTS or for other rapid diagnostic tests with a similar design. Researchers interested in quantitating lateral flow test results should note this issue.

In conclusion, this study has clearly shown that qFTS ratios closely correspond to CFA levels as measured by ELISA. Although further optimization is certainly possible, the spectrodensitometry method used in this study provides an important proof of principle. Spectrodensitometric reading of FTS provides a field-friendly method for rapidly measuring CFA levels that are related to adult filarial worm counts, which is much more convenient and less expensive than previously described methods. Additional work is needed to confirm and extend these preliminary observations for filariasis and to further explore the use of spectrodensitometry for measurement of other analytes detected by POC tests. While POC tests are already widely used in wealthy countries, they are increasingly being used in resource-limited settings where physicians and public health workers do not have ready access to high-quality clinical laboratories.

## Figures and Tables

**Table 1:** Spearman's rank correlation coefficients between qFTS ratios, vFTS scores, and Og4C3 ELISA optical density values

	vFTS	*P* values	Og4C3	*P* values
qFTS				
Whole population	0.9351	< 0.001	0.9102	< 0.001
Pre-MDA	0.9317	< 0.001	0.9043	< 0.001
Post-MDA	0.9381	< 0.001	0.9020	< 0.001
OD450 Og4C3				
Whole population	0.9140	< 0.001	–	–
Pre-MDA	0.9156	< 0.001	–	–
Post-MDA	0.9047	< 0.001	–	–

ELISA = enzyme-linked immunosorbent assay; MDA = mass drug administration; vFTS = visual Filariasis Test Strip; qFTS = quantitative Filariasis Test Strip.

**Table 2:** The results of a ROC analysis to determine the cutoff for the qFTS ratios using vFTS results as the reference standard

	Sensitivity	Specificity	Correctly classified	PPV	NPV	PLR	NLR
qFTS						
0.27	96.0	80.0	94.1	97.1	74.1	4.8	0.05
0.30	94.9	84.0	93.6	97.7	70.0	5.9	0.06
0.35	91.0	84.0	90.1	97.6	56.8	5.7	0.11
0.40	90.4	88.0	90.1	98.2	56.4	7.5	0.11
0.43	89.8	92.0	90.1	98.8	56.1	11.2	0.11

NLR = negative likelihood ratio; NPV = negative predictive value; PPV = positive predictive value; PLR = positive likelihood ratio; ROC = receiver operating characteristic; vFTS = visual Filariasis Test Strip; qFTS = quantitative Filariasis Test Strip ratios.

Sensitivity, specificity, correctly classified, PPV, and NPV results are expressed as percentages.

**Table 3:** Comparison of vFTS scores and Og4C3 ELISA results using different ELISA cutoff criteria

		S2 as cutoff		S3 as cutoff	
		Negative	Positive	Negative	Positive
vFTS scores	0	23	1	23	1
	1	23	29	42	10
	2	0	38	4	34
	3	0	76	0	76

vFTS = visual Filariasis Test Strip; ELISA = enzyme-linked immunosorbent assay.

The optical density values are obtained with standards S2 and S3, respectively.
